# Assessing Quality of Life Among Women with Urinary Incontinence—Medical, Psychological, and Sociodemographic Determinants

**DOI:** 10.3390/jcm14144839

**Published:** 2025-07-08

**Authors:** Beata Pilarska, Katarzyna Strojek, Agnieszka Radzimińska, Magdalena Weber-Rajek, Piotr Jarzemski

**Affiliations:** 1 Department of Urology, Collegium Medicum in Bydgoszcz, Nicolaus Copernicus University, 85-067 Bydgoszcz, Poland; piotr.jarzemski@cm.umk.pl; 2 Department of Physiotherapy, Collegium Medicum in Bydgoszcz, Nicolaus Copernicus University, 85-067 Bydgoszcz, Poland; katarzyna.strojek@cm.umk.pl (K.S.); radziminska@cm.umk.pl (A.R.); m.weber@cm.umk.pl (M.W.-R.)

**Keywords:** urinary incontinence in women, quality of life

## Abstract

**Introduction**: Urinary incontinence (UI) is associated with uncontrolled urine leakage and is treated as a serious disability that prevents the fulfillment of life roles and negatively affects quality of life. Many women do not have knowledge about the nature of UI and treatment options, and the embarrassing nature of the disease makes it difficult to seek specialist care. The aim of this study was to assess quality of life among women with UI and how it affects various areas of their daily functioning. Defining factors that modify the impact of UI on quality of life can provide prognostic information about functional limitations, which will facilitate the rapid implementation of preventive and therapeutic measures. **Methods**: This study included 158 women with UI. Patients were asked to complete a set of questionnaires, including the Questionnaire for Urinary Incontinence Diagnosis (QUID), Revised Urinary Incontinence Scale (RUIS), King’s Health Questionnaire (KHQ), Acceptance of Illness Scale (AIS), Inventory for Measuring Coping with Stress (Mini-COPY), and Set of Scales for Self-Assessment of the relationship with a partner. **Results**: Based on the analyses, it was determined that women with MUI experienced a lower quality of life, greater limitations in daily activities, and greater physical limitations compared to women with UUI and SUI. There was a correlation between the severity of UI, the duration of the disease, the level of acceptance of the disease, the education level of the subjects, and quality of life in all areas of functioning. **Conclusions**: Numerous functional limitations and reduced quality of life have been observed among patients with UI. As part of UI management in clinical practice, it seems reasonable to include measures aimed at identifying patients who are likely to experience more severe consequences of UI so that they can receive targeted care.

## 1. Introduction

Urinary incontinence (UI) is associated with uncontrolled urine leakage. According to data presented at the first EU Continence Health Summit in Brussels, about 55–60 million Europeans suffer from continence-related health problems [1]. UI affects every age group, and its incidence increases with age. About 10% of all adult women report one incident of UI per week, and sporadic leaks already occur in about 25–45% of women [1]. These numbers are successively increasing year after year, and incontinence is not only a health and financial burden for the individual but also carries social (including absenteeism from work, isolation) and economic consequences (including increased medical and non-medical costs associated with diagnosis and treatment). The specificity and inconvenience of the symptoms result in changes in habits among women with incontinence and force them to adopt certain behaviors to cope with the symptoms. The embarrassing nature of the condition hinders early implementation of diagnostic and therapeutic measures. The lack of knowledge among women about UI and available treatment leads them to choose ineffective remedies, resulting in a reduced sense of happiness and well-being.

Despite the high prevalence of UI, providing comprehensive care for a woman with UI is still a major challenge. The treatment of urinary dysfunction and its psychosocial consequences requires the involvement of interdisciplinary treatment teams. The authors believe that identifying factors that modify the impact of UI on quality of life can provide predictive information about functional limitations, which is crucial for guiding personalized and appropriate treatment of incontinence.

The main objective of this study was to assess quality of life among women with incontinence and its effects in various functional areas. Among the determinants modifying the impact of UI on well-being, three categories were distinguished: medical (type of UI, severity of UI, duration of UI), psychological (acceptance of the disease, how to cope with stress, quality of relationship with partner), and sociodemographic.

## 2. Materials and Methods

### 2.1. Study Population

Out of the 165 women who applied, the study group consisted of 158 women with different types and severities of UI who applied to the Department of Physiotherapy, the Department of Geriatrics, and the Department of Urology of the Medical College in Bydgoszcz to participate in the project “Application of non-invasive methods of treatment of pelvic floor muscle disorders”. This study was conducted in accordance with the guidelines of the Declaration of Helsinki and with the approval of the local Bioethics Committee. Inclusion criteria for this study were as follows: age over 18, voluntary consent to the study, women with urinary incontinence, and the ability to move independently. Exclusion criteria were contraindications to exercise and the proposed therapy, dementia and cognitive impairment, deafness, blindness, pregnancy, cancer, and an inability to determine the type of UI. All patients gave informed written consent to participate in the study. Prior to enrollment in the study, all patients attended a medical consultation, during which the type of UI and its severity, as well as fulfilment of the inclusion and exclusion criteria, were verified.

This study excluded 7 women whose UI symptoms were nonspecific and required further diagnosis. Patients completed questionnaires (validated and recommended for people with UI) before receiving any of the project’s therapies.

### 2.2. Research Tools

- Questionnaire for Urinary Incontinence Diagnosis (QUID). This questionnaire is recommended for differentiating types of incontinence. It consists of six questions about the circumstances of incontinence and urgency to urinate. The first three questions qualify Stress Urinary Incontinence (SUI), and the other three qualify Urge Urinary Incontinence (UUI). Responses are summed additively, with scores ranging from 0 to 15 points. Optimal cutoff values of (questions 1, 2, 3) ≥ 4 points and (questions 4, 5, 6) ≥ 6 points identify women with SUI and UUI, respectively. Women are classified as having Mixed Urinary Incontinence (MUI) if both scores are above the optimal cutoff values [2,3].
- Revised Urinary Incontinence Scale (RUIS). This scale is designed to assess the severity of urinary incontinence. It consists of five questions, and each answer is scored accordingly. The score ranges from 0 to 16 points: 0–3 = no UI or very mild, 4–8 = mild severity of symptoms, 9–12 = moderate, and 13–16 = a severe form of UI [4].
- Acceptance of Illness Scale (AIS). This scale provides information about the patient’s level of acceptance of the disease. It consists of eight statements that address the limitations and difficulties associated with the disease. The score ranges from 8 to 40 points. The higher the score, the better the adaptation and the lower the discomfort from the disease [5].
- Inventory for Measuring Coping with Stress Mini-COPE: This questionnaire provides insight into typical ways of responding in difficult and stressful situations. The survey adopts a seven-factor scale structure, consisting of the following strategies: active coping, helplessness, support-seeking, avoidant behavior, turning to religion, acceptance, and humor [6].
- King’s Health Questionnaire (KHQ). This questionnaire is a recommended tool designed to assess the quality of life of women with urinary incontinence. It consists of 21 items that relate to different spheres of life: Part One: general health perception (KHQ1) and incontinence impact (KHQ2); Part Two: role limitations (KHQ3), physical limitations (KHQ4), social limitations (KHQ5), personal relationships (KHQ6), emotions (KHQ7), sleep/energy (KHQ8), and severity measures (KHQ9); Part Three: a scale containing ten different symptoms related to lower urinary tract dysfunction and the degree to which they bother the patient (KHQ10). Responses in Section 1 and Section 2 are scored from 0 (best quality of life) to 100 (worst quality of life). On the other hand, Section 3, when summed up, is scored from 0 (best quality of life) to 30 points (worst quality of life). The lower the score, the better the quality of life in each area of the KHQ. This study used quality-of-life scores in the nine functional areas of the KHQ1-KHQ9. Part three of the questionnaire provided information on the nature of the complaints necessary for medical verification [7].
- Set of Scales for Self-Assessment of the relationship with a partner. This includes three five-item scales: the Emotional Bond Self-Assessment Scale, the Sexual Bond Self-Assessment Scale, and the Relationship Self-Assessment Scale. One has to choose the response that most closely corresponds to their relationship in a given area [8].

## 3. Results

### 3.1. Characteristics of Study Group

The average age of the subjects was 61.4 ± 13.32 (minimum age: 26 years; maximum age: 85 years). Most women attended secondary education (52.5%) and higher education (24.7%). Most declared a relationship with a partner (63.3%). Most women were retired (60.1%), and 34.2% were economically active. The vast majority lived in a large city with a population of over 30,000 (73.4%) (Table 1).

### 3.2. Type of UI vs. Assessment of Quality of Life

Table 2 presents descriptive statistics for the measured variables, the results of the Kruskal–Wallis test used to determine the difference in KHQ domain scores between the UI groups, and the results of the post hoc test.

Statistically significant differences (*p* < 0.05) were observed in five domains between the groups. Women with SUI rated their health (KHQ 1) and sleep/energy (KHQ 8) significantly better (*p* = 0.001 for both) compared to the other groups.

Women with MUI reported a statistically significant greater impact of UI on their lives (KHQ 2, *p* = 0.004), the most limitations in daily activities (KHQ 3, *p* = 0.018), and the most physical limitations (KHQ 4, *p* = 0.009) compared to the other groups.

### 3.3. UI Severity and Quality-of-Life Assessment

Table 3 shows the descriptive statistics for the measured variables, the results of the Kruskal–Wallis test used to determine the difference in the scores of the KHQ areas between the UI severity groups, and the results of the post hoc test.

Statistically significant differences (*p* < 0.05) were observed between UI severity groups in all areas of functioning. Quality-of-life scores in all domains in the severe UI severity group differed significantly from the other groups.

### 3.4. UI Duration and Quality of Life

The average duration of UI was 7.3 ± 3.58 years; the minimum time was 0.5 years, and the maximum time was 48 years. Table 4 shows descriptive statistics and Spearman’s rank correlation test results on the comparison of UI duration and quality of life.

A statistically significant positive correlation (*p* < 0.05) was observed between UI duration and quality-of-life scores in all domains of the KHQ.

### 3.5. Level of Acceptance of Disease and Quality of Life

Table 5 presents the results of Spearman’s rank correlation test regarding the comparison of the level of acceptance of the disease and the results of assessing quality of life.

A statistically significant negative correlation (*p* < 0.05) was observed between the disease acceptance level and quality-of-life scores in all KHQ domains.

### 3.6. Strategies to Deal with Stress and Quality of Life

Table 6 presents the results of Spearman’s rank correlation test regarding the comparison of ways to cope with stress and assessment of quality of life.

A statistically significant negative correlation (*p* < 0.05) was observed between active coping and six domains: the impact of bladder problems on life (KHQ 2, *p* = 0.008; Rho = −0.209), restrictions on everyday activities (KHQ 3, *p* = 0.013; Rho = −0.198), social restrictions (KHQ5, *p* = 0.005; Rho = −0.220), personal life (KHQ 6, *p* = 0.002; Rho = −0.314), emotions (KHQ 7, *p* < 0.001; Rho = −0.276), and sleep/energy (KHQ 8, *p* = 0.011; Rho = −0.202). Additionally, a statistically significant positive correlation (*p* < 0.05) was found between helplessness and emotion (KHQ 7, *p* = 0.026; Rho = 0.177) as well as sleep/energy (KHQ 8, *p* = 0.029; Rho = 0.174); a statistically significant negative correlation was found (*p* < 0.05) between seeking support and three domains: social restrictions (KHQ 5, *p* = 0.002; Rho = −0.244), personal life (KHQ 6, *p* = 0.001; Rho = −0.324), and measures of severity (KHQ 9, *p* = 0.041; Rho = −0.163), and a statistically significant positive correlation was found (*p* < 0.05) between an avoidant attitude and sleep/energy (KHQ 8, *p* = 0.038; Rho = 0.165).

### 3.7. Satisfaction with a Relationship with a Partner and Quality of Life

We adopted satisfaction with their relationship with their partner as a measure in this study, including emotional bond, sexual bond, and general assessment of the relationship. A total of 63% of women declared that they were in relationship. The average duration of the relationship was 32.9 ± 14.5 years. The minimum length was 2 years, and the maximum length was 60 years. In total, 44% of women declared that they were sexually active.

Table 7 presents the results of Spearman’s rank correlation test of the comparison of satisfaction with their relationship with their partner and quality-of-life assessment scores.

Emotional bond: A statistically significant negative correlation (*p* < 0.05) was observed between their emotional assessment and health assessment (KHQ 1, *p* = 0.001; Rho = −0.317), social restrictions (KHQ 5, *p* = 0.026; Rho = −0.224), and personal life (KHQ 6, *p* = 0.049; Rho = −0.222).

Self-assessment of sexual bond: A statistically significant negative correlation (*p* < 0.05) was observed between the assessment of sexual bond and health assessment (KHQ 1, *p* = 0.025; Rho = −0.214) as well as sleep/energy (KHQ 8, *p* = 0.017; Rho = −0.228). A general assessment of their relationship with their partner: A statistically significant negative correlation (*p* < 0.05) was observed between the general assessment of their relationship with their partner and health assessment (KHQ 1, *p* = 0.002; Rho = −0.301).

### 3.8. Sociodemographic Factors and Quality of Life

Table 8 presents the results of Spearman’s rank correlation test comparing age, education, and KHQ domain scores, as well as the results of the Kruskal–Wallis test used to determine the differences in KHQ domain scores between personal situation, professional status, and place of residence.

Age: A statistically significant positive correlation (*p* < 0.05) was observed between age and health assessment (KHQ1, *p* = 0.003; Rho = 0.233) and sleep/energy (KHQ8, *p* = 0.035; Rho = 0.168), and a negative correlation was observed with symptom severity (KHQ9, *p* = 0.018; Rho = −0.189).

Education: A statistically significant negative correlation (*p* < 0.05) between education and the assessment of quality of life in all domains was observed. Single significant differences in quality-of-life assessment between groups based on professional status, place of residence, and personal situation were observed.

## 4. Discussion

The consequences of incontinence according to the KHQ were observed in every sphere of the patients’ lives. Women with MUI declared the lowest quality of life in all areas of functioning, which is in line with the available literature [9]. Other researchers have proven that patients with MUI are more exposed to a moderate or serious influence of incontinence on their quality of life compared to women with SUI [10]. They also pay attention to significant differences between groups and restrictions in the social, personal, and emotional spheres, but this was not confirmed in our study. Our study findings demonstrate that, compared to women with UUI and SUI, women with MUI rated the impact of incontinence on life, role limitations (cooking, cleaning, shopping, professional activity), and physical limitations (walking, playing sports, traveling) significantly worse. The adverse impact of incontinence on traveling has also been noted by other researchers who have confirmed that the lack of access to toilets in new places and the inability to predict urine leakage caused fear and anxiety not only in women with MUI but also in those with UUI [11]. In our study, we observed that women with MUI rated their health significantly worse than women with SUI. Similar results were also obtained by other researchers who found that the perception of health among women with MUI was lower than in women with SUI and UUI [12]. Women with MUI in our own study also reported significantly worse sleep quality compared to women with SUI, similar to other studies [13,14].

The results obtained suggest that women with MUI are affected most by functional limitations and a lower quality of life.

Taking into account the severity of UI, our study observed that severe UI most significantly impaired life in all areas of functioning. We also found a similar conclusion to other researchers who stated that the severity of UI is the most important prognostic factor of reduced quality of life (QoL) and affects the search for specialist help [15,16,17]. It seems right that every patient with severe or moderate incontinence should be assessed in terms of quality of life.

The literature also presents different results that suggest that the influence of UI on the perception of well-being is more qualitative than quantitative. Even a slight uncontrolled leakage of urine causes very serious changes in the mood of patients, and the later severity of symptoms changes feelings in a slight way [18]. It is important to consider that patients may perceive their symptoms differently, which can influence how the impact of UI on their quality of life is reported and interpreted.

Another factor affecting the well-being of patients with UI is the duration of the disease. In a study conducted among Korean women, the researchers pointed to the relationship between the degree of severity of UI and the duration of illness [19]. Women with moderate and serious UI had suffered for much longer than those with mild UI. They reported more restrictions, a perceived inferior quality of life, and greater sexual dysfunction. A longer duration of UI is very often associated not only with a lower QoL but also with poorer perception of health [20]. In a study conducted among the inhabitants of Naples, the researchers confirmed that people who have been sick for more than 5 years have a more advanced form of UI and a lower sense of well-being [21].

Our study similarly determined that the longer women had UI, the lower their reported quality of life in all domains.

Our study also established that greater acceptance of the disease correlated with better quality of life. These observations are in line with available publications that indicate that the level of acceptance of the disease is an important prognostic factor for the quality of life of patients. The greater their acceptance of their disease, the less negative emotions they experience and the greater their involvement in actively improving their own health [22]. Acceptance of the disease helps patients to benefit from the available privileges (specialist care, reimbursement of drugs and incontinence care, various forms of support and therapy, etc.), restores a sense of agency, and increases the chances of overcoming the restrictions related to the disease.

Life with UI is associated with making everyday efforts to deal with the symptoms of incontinence; in order to maintain an appropriate level of functioning, women with UI must employ various mechanisms to cope with difficult situations. With the help of coping mechanisms, they can retain their identity and life competences. Women who adopt strategies that do not rely on religion, distraction, or denial have a high quality of life [23]. Attitudes aimed at masking or preventing the occurrence of symptoms may result in physical, mental, and social complications. The consequence of using such strategies is social isolation and increased susceptibility to depression and panic anxiety [24].

Our study found that the more often women used active coping mechanisms, the better they assessed their quality of life in six domains: personal relationships, incontinence impact, role limitations, social limitations, emotions, and severity measures. These findings are consistent with other studies showing that active strategies are associated with lower levels of stress related to illness, improved quality of life, and better overall well-being [25,26,27].

The opposite relationship was discovered in the case of increasing helplessness, which manifested itself in the cessation of action, self-blame, or the use of psychoactive substances. The co-occurrence of helplessness with increased sleep deprivation and greater emotional disorders in the form of depression, concern, and self-esteem disorders was noted. Other researchers also confirm this correlation [26,27,28]. They argue that helplessness can significantly worsen quality of life among women with UI, leading to symptom worsening and the development of anxiety.

Urinary incontinence is not only a physiological problem, but also a psychological and social one. It is associated with feelings of shame, lowered self-esteem, and withdrawal from family and social life. Support from loved ones, acceptance from those around them, and help from medical staff can have a positive impact on quality of life, decisions about treatment, and motivation to take action [26,29,30]. Our study also confirmed that seeking support was associated with improved scores in three areas of the KHQ: personal life, social limitations, and severity measures.

Noteworthy is the relationship between avoidance and sleep comfort. Good sleep is an indicator of good functioning in various fields. It not only affects health and well-being, but also makes it easier to actively engage in life and deal with adversities [31]. Nocturia and unwanted urine leakage during sleep, as researchers indicate, seriously deteriorate the quality of life of patients with UI [32]. Permanent exhaustion after sleepless nights reduces vitality and any desire to cope with the disease. It is therefore important that healthcare staff ask patients about the impact of their bladder problems on their sleep and QoL and provide support and treatment options to address these negative health effects. People with nocturia are more vulnerable to experiencing helplessness and powerlessness when struggling with the disease.

During the disease, many people use religious beliefs and practices to relieve stress and maintain a sense of control and meaning in life. Religion stimulates positive emotions, improves quality of life, and provides resources to cope with difficult situations. Numerous studies confirm the positive relationship between religion and mental health, health attitudes, and treatment [33,34]. The relationship between a return to religion and quality of life was not confirmed in our study.

The most frequently chosen strategy among respondents was acceptance. This involves recognizing the fact that UI is a part of your life and you have to adapt to this situation. In our study, as the level of acceptance increased, so did assessment scores for quality of life, except for health and sleep. However, this correlation did not remain significant when assessing the relationship among these factors. On the other hand, the benefits of using this strategy have been confirmed by other researchers who prove that acceptance correlates with better quality of life [20].

A lack of significance was observed with humor, the least often chosen strategy. Undoubtedly, however, a sense of humor helps create distance between yourself and the situation, making it easier to adapt to changes, which can be helpful in dealing with stress.

Perceived happiness well-being are also affected by satisfaction with a close relationship with another person. It was established in our study that feeling a greater emotional bond with a partner led to improvements in the assessment of general health, functioning in social contexts, and personal relationships. Better health assessment also co-occurred with better general assessment of their relationship with a partner and feeling a greater sexual bond. These results are consistent with the findings of other researchers who also claim that people who are in a successful marriage tend to have better physical and mental health and lower mortality than lonely or divorced people [35,36]. People with good physical health more frequently report being in a relationship with a partner and experiencing satisfaction with their sexual relationship; they are also more likely to have sexual intercourse [37].

Numerous publications have shown that fears of unwanted UI during sexual intercourse reduce sexual activity [4,20,23,38,39]. The discomfort caused by UI during sexual intimacy with a partner may influence women’s decisions to abstain from sex and avoid interpersonal contact with the opposite sex [40]. Bladder problems and UI may adversely affect a couple’s marital relationship and sexual activity [41]. Our study showed a similar result, with UI being the reason for avoiding sexual intercourse in 30.1%, reducing the feeling of fulfillment and satisfaction in 35.2%, and hindering sexual intercourse in 37.5%.

In the literature, we also find that sleep quality is affected by sexual functioning. A longer sleep duration is significantly correlated with higher sexual desire, increased commitment to sexual activity, and improved sexual relationships between partners. Sufficient sleep is important for mental and physical health, as well as for proper functioning in the intimate sphere [42,43].

Our study showed that patients experiencing a weak sexual bond with their partner rated their quality of their sleep the lowest compared to all groups. Chronic nocturia and night problems related to incontinence cause chronic fatigue, which could affect their relationship with their partner. The authors agree that one should take into account the assessment of their relationship with their partner when planning UI care for a patient, as it provides information about how they cope with UI. Early diagnosis of the problem could prevent disharmony in a relationship and contribute to the implementation of proper support for partners.

Among the sociodemographic factors modifying the influence of UI on perceived well-being is the age of the respondents. In our study, it was observed that with increased age, the perception of general health among the respondents decreased and perceived quality of sleep decreased, while severity measures improved. In the literature, some studies suggest that older patients have greater acceptance of their ailments because they are expected as the body ages. Thus, reduced health or weaker psychosocial functioning does not significantly reduce quality of life in this area. The situation is different among young people, as they expect vitality and good health. Each deviation from the socially imposed norm meets disapproval and manifests itself in a lower sense of well-being [8].

In our study, we also found that attending higher education co-occurred with better quality of life in all areas of functioning. Similar observations can be found in the available literature. Educated women pay more attention to their own health, are more likely to adopt pro-health attitudes, and lead a healthy lifestyle [44]. The right approach to UI is also influenced by the knowledge that a woman has about the disease and the possibilities of treatment. Women who receive incorrect information about their bodies at home or in their community are unable to talk about problems related to incontinence, which makes it difficult for them to seek treatment [45].

## 5. Conclusions

In this study, we observed a relationship between medical conditions (type of UI, severity of UI, duration of UI), psychological conditions (acceptance of disease, coping with stress, quality of relationship with partner), and sociodemographic conditions (age, education) and quality of life among women with UI.

**Figure 1:**
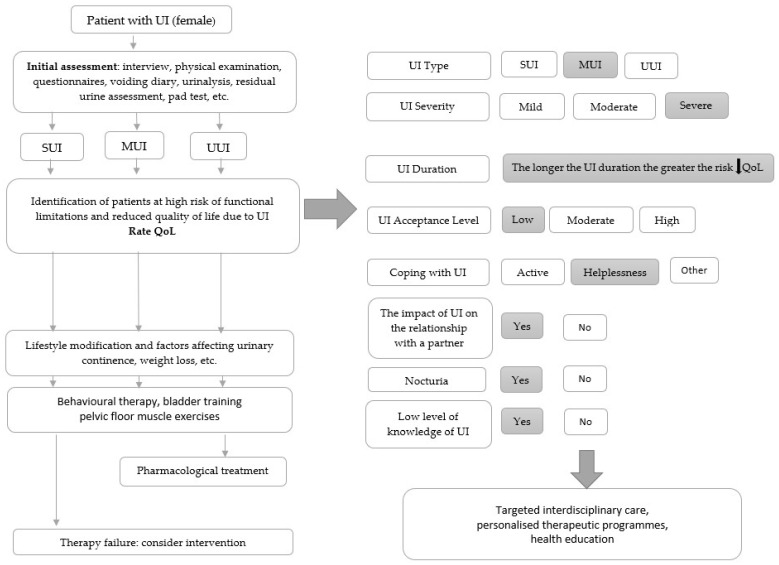
A diagram of the treatment procedure of a patient (female) with UI in clinical practice. UI—urinary incontinence; SUI—Stress Urinary Incontinence; MUI—Mixed Urinary Incontinence; UUI—Urge Urinary Incontinence; QoL—quality of life.

By observing the numerous functional limitations and reduced quality of life reported by patients with UI, it seems reasonable to include measures aimed at identifying patients who are likely to experience more severe consequences of UI as part of UI management in clinical practice so that they can receive targeted care, particularly those with MUI, severe UI, a longer duration of disease, a low level of acceptance, feelings of helplessness toward UI, experiencing negative effects of UI on their relationships, and nocturia (Figure 1).

## 6. Limitations

This study has several limitations. It was carried out among women looking for specialist help in connection with incontinence; therefore, the group may not be representative of the general population. The lack of a control group made it impossible to compare quality of life with a healthy population without bladder-related ailments. UI treatment methods that patients used before enrolling in this study were also not included, nor were comorbidities and medications that may affect health-related quality of life.

## Figures and Tables

**Table 1:** Group characteristics.

Feature (Variable)	*n*	%
Age (years)		
to 50	33	20.9
51–60	27	17.1
61–70	55	34.8
>70	43	27.2
Education		
elementary	6	3.8
vocational	30	19
secondary	83	52.5
higher education	39	24.7
Personal situation		
in a relationship	100	63.3
single	31	19.6
widow	27	17.1
Professional status		
professionally active	54	34.2
lack of employment	9	5.7
pension/disability pension	95	60.1
Place of residence		
country	18	11.4
small town (to 30,000)	24	15.2
big city (>30,000)	116	73.4

*n*—group size; %—percentage.

**Table 2:** Type of UI and quality-of-life assessment.

UI Type *n*	SUI 49	UUI 53	MUI 56	Kruskal–Wallis Test	Post Hoc Test				
KHQ	M	M	M	*p*		SUI	UUI	MUI	
KHQ1	27.55	45.28	45.54	0.001	SUI		<0.001	<0.001	*p*
					UUI	<0.001		0.950	
					MUI	<0.001	0.950		
KHQ2	50.34	54.09	68.45	0.004	SUI		0.536	0.003	*p*
					UUI	0.536		0.015	
					MUI	0.003	0.015		
KHQ3	36.73	38.05	54.46	0.018	SUI		0.848	0.009	*p*
					UUI	0.848		0.014	
					MUI	0.009	0.014		
KHQ4	48.64	45.28	62.20	0.009	SUI		0.570	0.021	*p*
					UUI	0.570		0.003	
					MUI	0.021	0.003		
KHQ5	22.79	27.36	36.61	0.108	n/a				
KHQ6	29.29	33.51	36.46	0.687	n/a				
KHQ7	32.65	37.53	41.67	0.327	n/a				
KHQ8	24.15	45.91	44.35	0.001	SUI		<0.001	<0.001	*p*
					UUI	<0.001		0.761	
					MUI	<0.001	0.761		
KHQ9	53.57	49.37	58.48	0.143	n/a				

*n*—group size; M—mean; *p*—level of statistical significance; UI—urinary incontinence; SUI—Stress Urinary Incontinence; MUI—Mixed Urinary Incontinence; UUI—Urge Urinary Incontinence; KHQ—King’s Health Questionnaire; n/a—not applicable.

**Table 3:** Severity UI and quality-of-life assessment.

Severity *n*	Weak (1) 8	Mild (2) 41	Moderate (3) 55	Severe (4) 54	Kruskal–Wallis Test	Post Hoc Test					
KHQ	M	M	M	M	*p*		(1)	(2)	(3)	(4)	
KHQ 1	34.38	33.54	37.04	48.18	0.009	(1)		0.921	0.747	0.095	*p*
						(2)	0.921		0.438	0.001	
						(3)	0.747	0.438		0.008	
						(4)	0.095	0.001	0.008		
KHQ 2	25.00	38.21	55.56	80.00	0.001	(1)		0.186	0.002	<0.001	*p*
						(2)	0.186		0.001	<0.001	
						(3)	0.002	0.001		<0.001	
						(4)	<0.001	<0.001	<0.001		
KHQ 3	18.75	19.11	40.43	68.18	0.001	(1)		0.975	0.052	<0.001	*p*
						(2)	0.975		0.001	<0.001	
						(3)	0.052	0.001		<0.001	
						(4)	<0.001	<0.001	<0.001		
KHQ 4	33.33	34.15	48.15	72.73	0.001	(1)		0.936	0.137	<0.001	*p*
						(2)	0.936		0.011	<0.001	
						(3)	0.137	0.011		<0.001	
						(4)	<0.001	<0.001	<0.001		
KHQ 5	12.50	10.16	23.15	51.82	0.001	(1)		0.830	0.320	<0.001	*p*
						(2)	0.830		0.027	<0.001	
						(3)	0.320	0.027		<0.001	
						(4)	<0.001	<0.001	<0.001		
KHQ 6	44.44	12.17	26.83	53.99	0.001	(1)		0.116	0.378	0.635	*p*
						(2)	0.116		0.050	<0.001	
						(3)	0.378	0.050		<0.001	
						(4)	0.635	<0.001	<0.001		
KHQ 7	19.44	20.60	33.95	56.16	0.001	(1)		0.913	0.162	<0.001	*p*
						(2)	0.913		0.019	<0.001	
						(3)	0.162	0.019		<0.001	
						(4)	<0.001	<0.001	<0.001		
KHQ 8	20.83	30.49	34.57	51.21	0.001	(1)		0.354	0.179	0.003	*p*
						(2)	0.354		0.465	<0.001	
						(3)	0.179	0.465		0.002	
						(4)	0.003	<0.001	0.002		
KHQ 9	15.63	35.98	54.78	71.97	0.001	(1)		0.011	<0.001	<0.001	*p*
						(2)	0.011		<0.001	<0.001	
						(3)	<0.001	<0.001		<0.001	
						(4)	<0.001	<0.001	<0.001		

*n*—group size; M—mean; *p*—level of statistical significance; KHQ—King’s Health Questionnaire.

**Table 4:** UI duration and quality of life.

UI Duration *n*	To 2 Years 50	3–5 Years 41	6–10 Years 35	Over 10 Years 32	Spearman’s Rho	
KHQ	M	M	M	M	Rho	*p*
KHQ1	32.50	43.29	44.29	42.19	0.174	0.029
KHQ2	43.33	57.72	68.57	69.79	0.349	<0.001
KHQ3	27.00	42.28	54.29	58.85	0.353	<0.001
KHQ4	37.33	51.22	60.48	68.23	0.388	<0.001
KHQ5	15.33	27.64	34.76	46.88	0.354	<0.001
KHQ6	21.30	34.29	36.23	41.67	0.230	0.024
KHQ7	25.33	41.46	38.41	50.35	0.291	<0.001
KHQ8	31.00	43.09	39.05	44.27	0.164	0.040
KHQ9	44.00	54.88	56.67	65.10	0.280	<0.001

*n*—group size; M—mean; *p*—significance level; Rho—Spearman’s correlation coefficient; KHQ—King’s Health Questionnaire.

**Table 5:** The correlation of the results of the level of acceptance of the disease.

KHQ	Rho	*p*
KHQ 1	−0.244	0.002
KHQ 2	−0.291	<0.001
KHQ 3	−0.430	<0.001
KHQ 4	−0.407	<0.001
KHQ 5	−0.390	<0.001
KHQ 6	−0.463	<0.001
KHQ 7	−0.403	<0.001
KHQ 8	−0.318	<0.001
KHQ 9	−0.434	<0.001

*p*—significance level; Rho—Spearman’s correlation coefficient; KHQ—King’s Health Questionnaire.

**Table 6:** Stress coping strategies and quality of life.

MINI-COPE	Active Coping		Helplessness		Seeking Support		Avoidant Behavior		Turn to Religion		Acceptance		Sense of Humor	
KHQ	Rho	*p*	Rho	*p*	Rho	*p*	Rho	*p*	Rho	*p*	Rho	*p*	Rho	*p*
KHQ 1	−0.092	0.252	0.090	0.259	−0.109	0.172	0.026	0.749	0.012	0.883	0.052	0.517	−0.056	0.487
KHQ 2	−0.209	0.008	0.062	0.436	−0.098	0.219	0.010	0.905	0.044	0.583	−0.067	0.400	−0.031	0.700
KHQ 3	−0.198	0.013	0.120	0.134	−0.125	0.117	0.050	0.530	0.003	0.967	−0.065	0.415	−0.019	0.815
KHQ 4	−0.128	0.109	0.036	0.653	−0.146	0.068	−0.007	0.926	0.017	0.835	−0.077	0.337	−0.014	0.863
KHQ 5	−0.220	0.005	0.110	0.167	−0.244	0.002	0.009	0.913	0.034	0.670	−0.080	0.320	−0.071	0.376
KHQ 6	−0.314	0.002	0.104	0.314	−0.324	0.001	−0.029	0.782	−0.004	0.970	−0.104	0.313	−0.095	0.356
KHQ 7	−0.276	<0.001	0.177	0.026	−0.149	0.062	0.084	0.296	0.004	0.955	−0.096	0.230	−0.023	0.778
KHQ 8	−0.139	0.082	0.174	0.029	−0.003	0.968	0.165	0.038	0.092	0.251	0.089	0.267	−0.022	0.785
KHQ 9	−0.202	0.011	0.108	0.176	−0.163	0.041	0.023	0.774	0.070	0.381	−0.073	0.364	−0.015	0.851

*p*—significance level; Rho—Spearman’s correlation coefficient; KHQ—King’s Health Questionnaire.

**Table 7:** Satisfaction with their partner relationship and quality of life.

	Emotional Bond with Their Partner		Sexual Bond with Their Partner		Self-Assessment of Their Relationship with Their Partner	
KHQ	Rho	*p*	Rho	*p*	Rho	*p*
KHQ 1	−0.317	0.001	−0.214	0.025	−0.301	0.002
KHQ 2	0.050	0.622	0.142	0.141	0.068	0.502
KHQ 3	0.054	0.598	0.076	0.432	0.077	0.451
KHQ 4	0.027	0.791	0.102	0.293	0.084	0.407
KHQ 5	−0.224	0.026	−0.146	0.130	−0.164	0.104
KHQ 6	−0.222	0.049	−0.185	0.099	−0.123	0.278
KHQ 7	−0.057	0.577	0.026	0.785	−0.023	0.819
KHQ 8	−0.179	0.077	−0.228	0.017	−0.133	0.188
KHQ 9	−0.167	0.099	−0.103	0.285	−0.162	0.109

*p*—significance level; Rho—Spearman’s correlation coefficient; KHQ—King’s Health Questionnaire.

**Table 8:** Sociodemographic variables and quality of life.

	Age		Education		Personal Situation		Professional Status		Place of Residence	
KHQ	Rho	*p*	Rho	*p*	H	*p*	H	*p*	H	*p*
KHQ 1	0.233	0.003	−0.280	<0.001	0.431	0.806	14.327	0.001	0.167	0.920
KHQ 2	−0.135	0.092	−0.261	0.001	1.097	0.578	3.523	0.172	2.101	0.350
KHQ 3	−0.100	0.210	−0.315	<0.001	0.023	0.989	0.920	0.631	2.740	0.254
KHQ 4	−0.140	0.079	−0.251	0.001	2.429	0.297	3.890	0.143	2.636	0.268
KHQ 5	0.041	0.609	−0.369	<0.001	2.047	0.359	2.314	0.314	0.066	0.967
KHQ 6	0.029	0.779	−0.298	0.003	9.001	0.011	0.850	0.654	0.076	0.962
KHQ 7	−0.130	0.103	−0.297	<0.001	0.546	0.761	4.664	0.097	0.725	0.696
KHQ 8	0.168	0.035	−0.412	<0.001	2.456	0.293	3.788	0.151	0.711	0.701
KHQ 9	−0.189	0.018	−0.201	0.011	1.619	0.445	9.604	0.008	6.020	0.049

*p*—significance level; Rho—Spearman’s correlation coefficient; H—value of the test statistic; KHQ—King’s Health Questionnaire.

## Data Availability

The datasets generated and/or analyzed during the current study are available from the corresponding author on reasonable request.
